# Algorithms for Fresnel Diffraction at Rectangular and Circular Apertures

**DOI:** 10.6028/jres.103.030

**Published:** 1998-10-01

**Authors:** Klaus D. Mielenz

**Affiliations:** Oakland, MD 21550

**Keywords:** algorithms, circular apertures, diffraction, Fresnel approximation, personal computers, radiometry, rectangular apertures, slits

## Abstract

This paper summarizes the theory of Fresnel diffraction by plane rectangular and circular apertures with a view toward numerical computations. Approximations found in the earlier literature, and now obsolete, have been eliminated and replaced by algorithms suitable for use on a personal computer.

## 1. Introduction

The basic theory of Fresnel diffraction at plane apertures was developed long ago [1,2] and is summarized in textbooks [3–6]. For apertures bounded by straight lines (rectangle, slit, half plane), the standard textbook solution in terms of complex Fresnel integrals^1^ is based on special but poorly documented transformations of coordinates. It is shown in this paper that such transformations cannot be performed accurately for apertures irradiated by arbitrarily located point sources. In the past, the numerical evaluation of the complex Fresnel integrals themselves has also been a problem, and thus previous discussions were confined to simplified special cases. Computational details were omitted and semi-quantitative methods (Cornu spiral) were used to describe the nature of diffraction at rectangular apertures.

In the case of circular apertures, the rigorous solution involves Lommel functions of two variables, which are defined as series expansions in Bessel functions and previously had to be evaluated by tedious manual calculations or approximations. For the most part, these approaches have been rendered obsolete by modern computer software. However, approximative methods are still useful for work on personal computers which involves large values of the configuration parameter *u* defined by Eq. (17a) of this paper. It is shown here that a previously used approximation by Focke [7] is inadequate for this purpose on account of its poor accuracy, but that an older approximation by Schwarzschild [8] gives excellent results.

As algorithms for the computation of Fresnel diffraction patterns on a personal computer have not been published, a compilation of such algorithms is presented in this paper. The underlying theory is stated for off-axis source points, so that the results can be applied to extended sources. For both types of aperture, the closed solutions obtained are paraxial approximations.

## 2. The Fresnel Diffraction Integral

The scalar wave function *U*(*P*) associated with diffraction at a plane aperture is customarily expressed by one of the Rayleigh-Sommerfeld integrals^2^,

$$U_{\text{RS}}^{p}(P)=-\frac{A_{\text{sph}}}{2\pi }\int dQ\frac{e^{ik(P_{0}Q+QP)}}{P_{0}Q\cdot QP}\left(ik-\frac{1}{P_{0}Q}\right)\frac{\partial P_{0}Q}{\partial n},$$ (1a)

$$U_{\text{RS}}^{s}(P)=-\frac{A_{\text{sph}}}{2\pi }\int dQ\frac{e^{ik(P_{0}Q+QP)}}{P_{0}Q\cdot QP}\left(ik-\frac{1}{QP}\right)\frac{\partial QP}{\partial n},$$ (1b)

or, alternatively, by the Kirchhoff integral,

$$U_{K}(P)=\frac{1}{2}(U_{\text{RS}}^{p}+U_{\text{RS}}^{s}).$$ (1c)

**Fig. 1:**
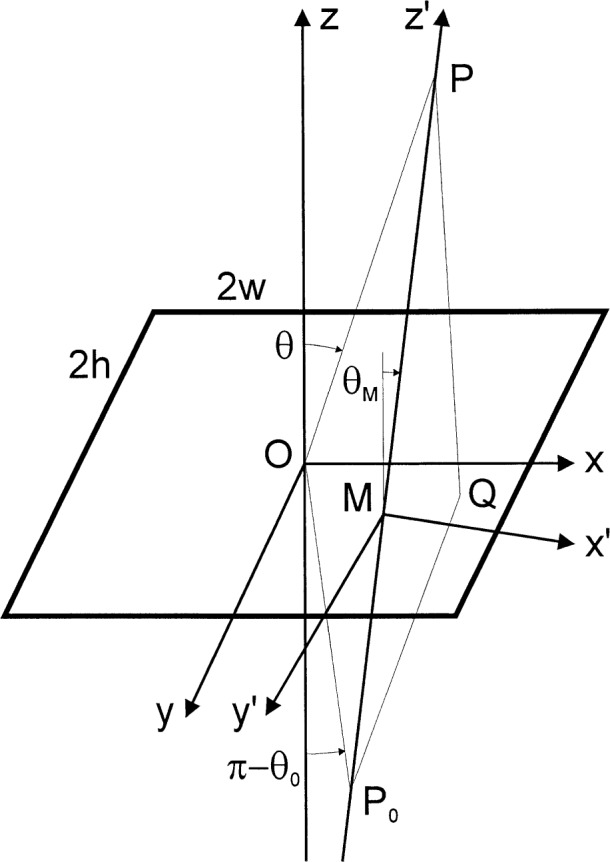
Notation for rectangular apertures.

**Fig. 2:**
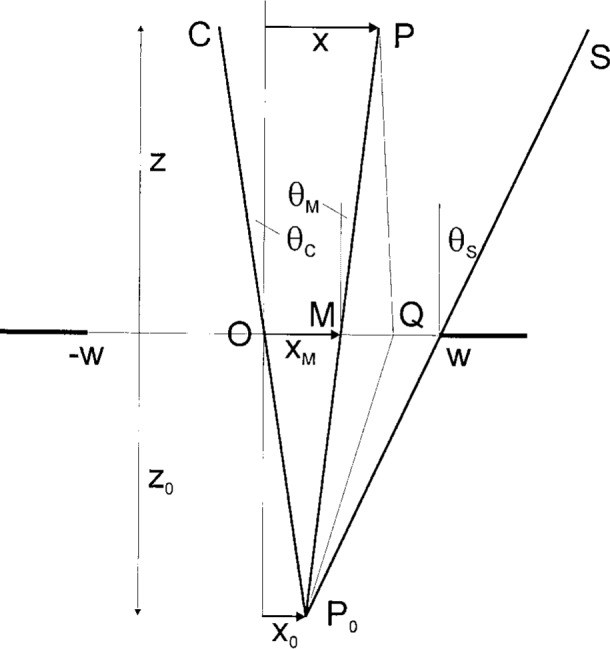
Notation for slits.

Here, as indicated in Figs. 1, 2, and 4, *P*_0_ is the location of a point source emitting a monochromatic spherical wave of amplitude *A*_sph_, circular wave number *k* = 2π/*λ Q* is a point in the aperture, d*Q* is the surface element at *Q*, ***n*** is the aperture normal pointing away from the source, and *P* is the point of observation.

In the Fresnel approximation the points *P*_0_ and *P* are located at finite distances which are large compared to the wavelength of light and the dimensions of the aperture. Therefore, it is assumed that

$$\frac{1}{P_{0}Q}<<k,\;\frac{1}{QP}<<k,$$ (2a)

and that the distances *P*_0_*Q* and *QP* and their normal derivatives do not vary appreciably inside the aperture. Thus they can be replaced, except in the rapidly oscillating exponential function in the integrand, by their values at an arbitrarily chosen reference point *O* inside the aperture. Under these conditions, the first Rayleigh-Sommerfeld integral Eq. (1a) may be written in the form

$$U_{\text{RS}}^{p}(P)\approx -\frac{ikA_{\text{sph}}}{2\pi }\frac{\partial P_{0}O}{\partial n}\frac{e^{ik(P_{0}O+OP)}}{P_{0}O\cdot OP}\int dQ\;e^{ik\Delta (Q)},$$ (2b)

where

$$\Delta (Q)=(P_{0}Q+QP)-(P_{0}O+OP),$$ (2c)

is a small quantity that can be expressed in approximate form. It is well known that, with

$$O=(0,0,0),\;P_{0}=(x_{0},y_{0},z_{0}),\;Q=(\xi ,\eta ,0),\;P=(x,y,z)$$ (2d)

expressed in cartesian coordinates, the required approximation for Δ(*Q*) is

$$\begin{array}{l} \Delta (Q)=-[(l-l_{0})\xi +(m-m_{0})\eta ]+\frac{1}{2r_{0}}[(\xi^{2}+\eta^{2})-\\ {\left(l_{0}\xi +m_{0}\eta \right)}^{2}]+\frac{1}{2r}[(\xi^{2}+\eta^{2})-{\left(l\xi +m\eta \right)}^{2}]+\epsilon (\xi ,\eta ), \end{array}$$ (2e)

where *ϵ* (*ξ*,*η*) is the residual error when terms of third and higher order in *ξ* and *η* are neglected. Here,

$$l_{0}=-\frac{x_{0}}{r_{0}},\;m_{0}=-\frac{y_{0}}{r_{0}},\;l=\frac{x}{r},\;m=\frac{y}{r}$$ (2f)

are the first and second direction cosines of the vectors ***P***_0_***O*** and ***OP***, and

$$r_{0}=\sqrt{x_{0}^{2}+y_{0}^{2}+z_{0}^{2}},\;r=\sqrt{x^{2}+y^{2}+z^{2}}$$ (2g)

are the distances *P*_0_*O* and *OP*. The corresponding value of the normal derivative^3^ in Eq. (2b) is

$$\frac{\partial P_{0}O}{\partial n}=-\frac{\partial r_{0}}{\partial z_{0}}=-\frac{z_{0}}{r_{0}}=-\cos\theta_{0}.$$ (2h)

We now have

$$U_{\text{RS}}^{p}(P)\approx \frac{ikA_{\text{sph}}\cos\theta_{0}}{2\pi r_{0}r}e^{ik(r_{0}+r)}\int dQe^{ik\Delta (Q)}.$$ (2i)

The corresponding forms of $U_{\text{RS}}^{s}(P)$ and *U*_K_(*P*) are essentially the same, except that –cos*θ*_0_ is replaced by cos*θ* and ½ (−cos*θ*_0_ + cos*θ*), respectively, where *θ* is the colatitude of the point of observation *P*. Because the diffracted light is confined to a narrow angular range about the central direction *P*_0_*Q* unless the aperture dimensions are extraordinarily small, these differences may be judged insignificant. As the Rayleigh-Sommerfeld solutions pertain to the respective cases of *p*- and *s*-polarization of the incident light, this implies that Fresnel diffraction is independent of polarization. The Kirchhoff solution has no definable meaning as far as polarization is concerned, but turns out to be equivalent to the Rayleigh-Sommerfeld solutions in the Fresnel approximation. A further solution, the Maggi-Rubinowicz transformation of Kirchhoff’s integral [3,4], is not suitable for computations of Fresnel diffraction patterns because it is singular at the boundary of the geometrical shadow.

In this paper, Eq. (2i) will be regarded as the basic form of the Fresnel diffraction integral and will be written as

$$U_{F}(P)=-U_{0}(P)\cos\theta_{0}\alpha_{F}(P),$$ (3a)

where

$$U_{0}(P)=A_{\text{sph}}\frac{e^{ik(r_{0}+r)}}{r_{0}+r}=\sqrt{E_{0}(P)}e^{ik(r_{0}+r)}$$ (3b)

is the geometrical field at the point of observation *P* according to Huygens’ principle,

$$\alpha_{F}(P)=-\frac{ik(r_{0}+r)}{2\pi r_{0}r}\int dQe^{ik\Delta (Q)}$$ (3c)

is the modification of the geometrical field by diffraction, *E*_0_(*P*) is the normally incident geometrical irradiance at *P*, and –cos*θ*_0_ is the inclination factor according to Lambert’s law.

The third- and fourth-order terms neglected in Eq. (2e) are

$$\begin{array}{l} \epsilon (\xi ,\eta )=-\frac{1}{2r_{0}^{2}}\{(l_{0}\xi +m_{0}\eta )[(\xi^{2}+\eta^{2})-{\left(l_{0}\xi +m_{0}\eta \right)}^{2}]\}\\ \;+\frac{1}{2r^{2}}\{(l\xi +m\eta )[(\xi^{2}+\eta^{2})-{\left(l\xi +m\eta \right)}^{2}]\}\\ \;-\frac{1}{8r_{0}^{3}}\{{\left(\xi^{2}+\eta^{2}\right)}^{2}-{\left(l_{0}\xi +m_{0}\eta \right)}^{2}[6(\xi^{2}+\eta^{2})\\ \;-5{\left(l_{0}\xi +m_{0}\eta \right)}^{2}]\}\\ \;-\frac{1}{8r^{3}}\{{\left(\xi^{2}+\eta^{2}\right)}^{2}-{\left(l\xi +m\eta \right)}^{2}[6(\xi^{2}+\eta^{2})\\ \;-5{\left(l\xi +m\eta \right)}^{2}]\}. \end{array}$$ (4a)

This shows that the error introduced by neglecting this term depends in a complicated manner on the geometrical parameters involved. Accordingly, it is difficult to assess its magnitude without considering specific cases. However, a few general comments are in order. Under ordinary circumstances, the direction cosines *l*_0_, *l*, … are small compared with unity, so that (*l*_0_*ξ* + *m*_0_*η*)^2^ and (*lξ* + *mη*)^2^ are much smaller than (*ξ*^2^ + *η*^2^), and then one finds^4^

$$\epsilon (\xi ,\eta )\approx -\frac{r_{0}^{3}+r^{3}}{8r_{0}^{3}r^{3}}{\left(\xi^{2}+\eta^{2}\right)}^{2}.$$ (4b)

Hence, the magnitude of *ϵ* (*ξ*,*η*) relative to the quadratic term of Eq. (2e) may be estimated as

$$\frac{2r_{0}r/\epsilon (\xi ,\eta )|}{\left(r_{0}+r)(\xi^{2}+\eta^{2}\right)}\approx \frac{\left(r_{0}^{3}+r^{3})(\xi^{2}+\eta^{2}\right)}{4r_{0}^{3}r^{2}(r_{0}+r)}<\frac{q_{\max}^{2}}{{\langle r\rangle }^{2}},$$ (4c)

where *q*_max_ is the maximum value of $\sqrt{\xi^{2}+\eta^{2}}$(e.g., the radius of a circular aperture) and 〈*r*〉 is an average of *r*_0_ and *r*. Accordingly, the relative error in Δ(*Q*) is inversely proportional to the square of the relative distance 〈*r*〉/*q*_max_. At a distance of ten aperture dimensions, it is on the order of 1 %.

## 3. Rectangular Aperture

### 3.1 General Theory (Fig. 1)

When applying the above equations to a rectangular aperture of width 2*w* and height 2*h*, it is customary to transform the global cartesian coordinates (*x*, *y*, *z*) assumed in Sec. 2 into local coordinates (*x*′, *y*′, *z*′) which depend on the locations of the points *P*_0_ and *P* and are chosen so that Eq. (3c) is separated into a product of independent Fresnel integrals in *ξ* and *η*. That is,

$$\alpha_{F}(P)\propto \int d\xi e^{ia\xi^{2}}\int d\eta e^{ib\eta^{2}}.$$ (5)

The first step in this transformation is to place the origin of the local coordinates at the point *M* where the straight line *P*_0_*P* intersects the aperture plane:

$$M=(x_{M},y_{M},0),\;x_{M}=\frac{x_{0}z-xz_{0}}{z-z_{0}},\;y_{M}=\frac{y_{0}z-yz_{0}}{z-z_{0}}.$$ (6a)

This gives

$$\begin{array}{c} {l_{0}}^{\prime }=\frac{x_{0}-x_{M}}{{r_{0}}^{\prime }}=l^{\prime }=\frac{x-x_{M}}{r^{\prime }},\\ {m_{0}}^{\prime }=\frac{y_{0}-y_{M}}{{r_{0}}^{\prime }}=m^{\prime }=\frac{y-y_{M}}{r^{\prime }},\\ {r_{0}}^{\prime }=\frac{-z_{0}}{\cos\theta_{M}},\;r^{\prime }=\frac{z}{\cos\theta_{M}}, \end{array}$$ (6b)

*θ_M_* being the angle indicated in Fig. 1. The linear term of Eq. (2e) now vanishes and we have

$$\begin{array}{l} \Delta (Q)=\frac{1}{2\rho^{\prime }}\{{\left(\xi -x_{M}\right)}^{2}+{\left(\eta -y_{M}\right)}^{2}\\ -{\left[{l_{0}}^{\prime }\left(\xi -x_{M}\right)+{m_{0}}^{\prime }\left(\eta -y_{M}\right)\right]}^{2}\}, \end{array}$$ (6c)

$$\rho^{\prime }=\frac{{r_{0}}^{\prime }r^{\prime }}{{r_{0}}^{\prime }+r^{\prime }}=\frac{-zz_{0}}{(z-z_{0})\cos\theta_{M}}.$$ (6d)

This result can be used in two ways to derive a final result.

#### 3.1.1 Paraxial Approximation

As mentioned in deriving Eq. (4b) above, the direction cosines *l*_0_′ and *m*_0_′ will be small if the points *P*_0_ and *P* are close to the *z*-axis of Fig. 1. To a first-order approximation in *θ_M_* we have *l*′_0_^2^, *m*′_0_^2^ « 1, so that the third term of Eq. (6c) can be omitted and Eq. (3c) leads directly to

$$\begin{array}{l} \alpha_{F}(P)\approx -\frac{ik}{2\pi \rho^{\prime }}\int_{-w}^{w}d\xi e^{ik{\left(\xi -x_{M}\right)}^{2}/2\rho^{\prime }}\int_{-h}^{h}d\eta e^{ik{\left(\eta -y_{M}\right)}^{2}/2\rho^{\prime }}\\ \;=-\frac{i}{2}[F(s_{+})-F(s_{-})][F(t_{+})-F(t_{-})], \end{array}$$ (7a)

where

$$s_{\pm }=\sqrt{\frac{k}{\pi \rho^{\prime }}}(\pm \;w-x_{M}),\;t_{\pm }=\sqrt{\frac{k}{\pi \rho^{\prime }}}(\pm \;h-y_{M}),$$ (7b)

and

$$F(s)=C(s)+iS(s)=\int_{0}^{s}d\sigma \;e^{i\pi \sigma^{2}/2}$$ (8)

is the complex Fresnel integral.

#### 3.1.2 Coordinate Transformation for Off-Axis Sources

According to textbooks, a rotation of coordinates may be necessary when the direction cosines *l*_0_′ and *m*_0_′ are too large to justify the paraxial approximation of Sec. 3.1.1, a rotation of coordinates may be necessary. The usual recommendation [3, 5] is to place the new *x*′-axis along the projection of the line *P*_0_*P* onto the aperture plane. This gives *m*_0_′ = 0, so that *α*_F_(*P*) does indeed assume the form stipulated by Eq. (5). However, the *x*′- and *y*′-axes so defined are not parallel to the edges of the aperture, and consequently the two integrals are not separable because the limits of the *ξ*-integral depend on *η*, and vice versa.

To overcome this difficulty, a different transformation is attempted in the following: The *z*′-axis is placed in the direction of the unit vector along the line *P*_0_*P*, so that *l*_0_′ = *m*_0_′ = 0 and Eq. (5) is again satisfied. The *x*′-axis is chosen so that its projection onto the aperture plane is parallel to the *ξ*-direction. The *y*′-axis is defined in the usual manner as ***y***′ = ***z***′ × ***x***′. Thus,

$$\left(\begin{array}{c} x^{\prime }\\ y^{\prime }\\ z^{\prime } \end{array}\right)=\left(\begin{array}{ccc} 1/W & 0 & -\cos\phi_{M}\tan\theta_{M}/W\\ -\sin\phi_{M}\cos\phi_{M}\sin\theta_{M}\tan\theta_{M}/W & W\cos\theta_{M} & -\sin\phi_{M}\sin\theta_{M}/W\\ \cos\phi_{M}\sin\theta_{M} & \sin\phi_{M}\sin\theta_{M} & \cos\theta_{M} \end{array}\right)\left(\begin{array}{c} x-x_{M}\\ y-y_{M}\\ z \end{array}\right)$$ (9a)

where *ϕ*_M_ and *θ*_M_ are the longitudes and colatitudes of *P*_0_ and *P* with respect to M,

$$\tan\phi_{M}=\frac{y-y_{0}}{x-x_{0}},\;\tan\theta_{M}=\frac{\sqrt{{\left(x-x_{0}\right)}^{2}+{\left(y-y_{0}\right)}^{2}}}{z-z_{0}},$$ (9b)

and

$$W=\sqrt{1+\cos^{2}\phi_{M}\tan^{2}\theta_{M}}.$$ (9c)

Accordingly, for *z* = 0,

$$\begin{array}{c} \xi^{\prime }=\frac{1}{W}(\xi -x_{M}),\\ \eta^{\prime }=W\cos\theta_{M}[\frac{-\sin\phi_{M}\cos\phi_{M}\tan^{2}\theta_{M}}{W^{2}}(\xi -x_{M})\\ +(\eta -y_{M})]. \end{array}$$ (9d)

Equation (9d) shows that *η*′ is still not independent of *ξ*. This was to be expected as it is not possible to rotate the *z*-axis and have orthogonal *x*- and *y*-axes which are both aligned with the aperture edges. Accordingly, the separation of integration limits is not complete unless the first term in the above expression for *η*′ is omitted—a first-order approximation in *θ_M_.* Then,

$$\begin{array}{c} \xi =\frac{1}{W}(\xi -x_{M}),\;\eta^{\prime }=W\cos\theta_{M}(\eta -y_{M})\;,\\ d\xi^{\prime }d\eta^{\prime }=\cos\theta_{M}d\xi d\eta . \end{array}$$ (10a)

and

$$\begin{array}{c} \alpha_{F}(P)=\\ -\frac{ik\cos\theta_{M}}{2\pi \rho^{\prime }}\int_{-w}^{w}d\xi e^{ik{\left(\xi -x_{M}\right)}^{2}/2W^{2}{\rho^{\prime }}^{2}}\int_{-h}^{h}d\eta e^{ikW^{2}\cos^{2}\theta {\left(\eta -y_{M}\right)}^{2}/2{\rho^{\prime }}^{2}}\\ =-\frac{i}{2}[F(s_{+}/W)-F(s_{-}/W)][F(W\cos\theta_{M}t_{+})\\ -F(W\cos\theta_{M}t_{-})] \end{array}$$ (10b)

where s_+_ and s_−_ are the same as in Eq. (7b), above. It should be noted that this result differs from the paraxial approximation only by the factors 1/*W* and *W* cos*θ_M_* in the arguments of the Fresnel integrals. Within the above approximation for *η*′ these factors are equal to unity, and thus Eq. (10b) appears to be no improvement over the paraxial approximation Eq. (7b) of Sec. 3.1.1. It follows that, for rectangular apertures, the coordinate transformations recommended in Refs. [3] and [5] are superfluous. To higher than first order in *θ_M_*, the *ξ*- and *η*-integrals remain inseparable and a closed solution for *α*_F_(*P*) is not possible.

#### 3.1.3 Evaluation of Fresnel Cosine and Sine Integrals

The use of Eqs. (7a) and (10b) is straightforward. An example is given in Sec. 3.2, below. It should be remembered that the variation of *α*_F_ (*P*) with *P* is implicit in Eqs. (7b) and (10b), in that *x_M_*, *y_M_*, *ϕ_M_* and *θ_M_* depend on the location of *P*. It should also be borne in mind that the point *M* of Fig. 1 will be outside the aperture when *P* lies in the geometric shadow. This can lead to values of *ξ* and *η* larger than assumed in Eq. (3a). For this reason, the computation of *α*_F_ (*P*) based on Eq. (7b) or (10b) must not be carried too far into the shadow region.

The only problem that may be encountered on a personal computer is that the Fresnel cosine and sine integrals defined by Eq. (8) are not usually included in standard software packages. For modest accuracy requirements, they can be computed from the equations quoted in Ref. [9],

$$\begin{array}{c} C(s)=\frac{1}{2}+f(s)\sin\left(\frac{\pi s^{2}}{2}\right)-g(s)\cos\left(\frac{\pi s^{2}}{2}\right),\\ C(-s)=-C(s), \end{array}$$ (11a)

$$\begin{array}{c} S(s)=\frac{1}{2}-f(s)\cos\left(\frac{\pi s^{2}}{2}\right)-g(s)\sin\left(\frac{\pi s^{2}}{2}\right),\\ S(-s)=-S(s), \end{array}$$ (11b)

$$f(s)=\frac{1+0.926\;s}{2+1.792\;s+3.104\;s^{2}}+\epsilon (s),\;s\geq 0,$$ (11c)

$$g(s)=\frac{1}{2+4.142\;s+3.492\;s^{2}+6.67\;s^{3}}+\epsilon (s),\;s\geq 0,$$ (11d)

where |*ϵ* (*s*)| ≤ 2 × 10^−3^. Accordingly, the following simple algorithm may be used:

1. Define *s*.
2. Let *s*′ = |*s*|.
3. Calculate *f*(*s*′), *g*(*s*′) from Eq. (11c,d).
4. Calculate *C*(*s*′), *S*(*s*′) from Eq. (11a,b).
5. If *s* < 0 let *C*(*s*) = − *C*(*s*′), *S*(*s*) = − *S*(*s*′). Else, let *C*(*s*) = *C*(*s*′), *S*(*s*) = *S*(*s*′).

If better accuracy is desired, this algorithm can be improved by using the method described in Ref. [10]. Alternatively, software for computing *C*(*s*) and *S*(*s*) in Fortran or C can be downloaded [11, 12].

### 3.2 Application to Slits (Fig. 2)

The rectangular aperture discussed so far is transformed into a slit of width 2*w* on setting *h* = ∞ in Eq. (7b).^5^ It may then also be assumed that the source is a long luminous line which is parallel to the slit and passes through the point *P*_0_ in Fig. 1, so that it will suffice to compute the diffraction pattern in the *xz*-plane shown in Fig. 2. With these assumptions we have *t*± = ± ∞, so that *F*(*t*_±_) = ± ½ (1 + i), [*F*(*t*_±_) − *F*(*t*_−_)] = 1 + i, and Eq. (10b) is reduced to

$$\begin{array}{c} \alpha_{F}(P)=\frac{1-i}{2}[F(s_{+})-F(s_{-})]\\ =\frac{1-i}{2}\{[C(s_{+})-C(s_{-})]+i[S(s_{+})-S(s_{-})]\}, \end{array}$$ (12a)

with *s* as defined by Eq. (7b) but assuming *y*_0_ = *y* = *y*_M_ = 0 so that Eq. (9b) is simplified to

$$\theta_{M}=\arctan\frac{x_{0}-x_{M}}{z_{0}},x=x_{M}+z\tan\theta_{M}.$$ (12b)

As the diffraction pattern is centered at and symmetrical about the geometrical source image *C* shown in Fig. 2, where

$$x_{M}=0,\;\theta_{M}=\theta_{C}=\arctan\frac{x_{0}}{z_{0}},\;x=x_{C}=\frac{x_{0}z}{z_{0}},$$ (12c)

it will also suffice to compute it for positive value values of *x_M_*, only. The computation is typically carried to a maximum value of *θ_M_* beyond the shadow boundary *S*, the latter being given by

$$x_{M}=w,\;\theta_{M}=\theta_{S}=\arctan\frac{x_{0}-w}{z_{0}},\;x=x_{S}=w+z\tan\theta_{S}.$$ (12d)

Accordingly, the following procedure may be used to evaluate the dependence of *α*_F_(*P*) on *x*:

1. Define a maximum (*x_M_*)_max_ and a step size Δ*x_M_* for *x_M_*.
2. Let *x_M_* = 0.
3. Compute *θ_M_* and *x* from Eq. (12b) and *s*_±_ from Eq. (7b). Use the algorithm of Sec. 3.1 to find *C*(*s*_±_) and *S*(*s*_±_). Compute *α*_F_(*P*) from Eq. (12a).
4. Let *x_M_* = *x_M_* + Δ*x_M_*. If *x_M_* < (*x_M_*)_max_, go to Step 3. Else, stop.

**Fig. 3:**
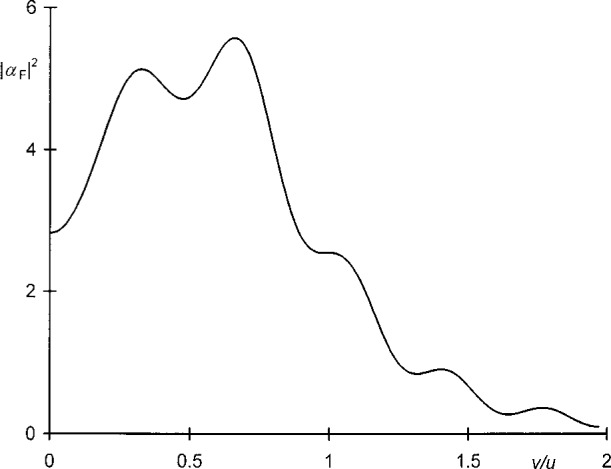
Relative irradiance, |*α*_F_(*u*, *v*)|^2^ vs *v*/*u*, for a slit. *w* = 1 mm, *z*_0_ = − 2.507 m, *z* = 1.140 m, *λ* = 639 nm.

**Fig. 4:**
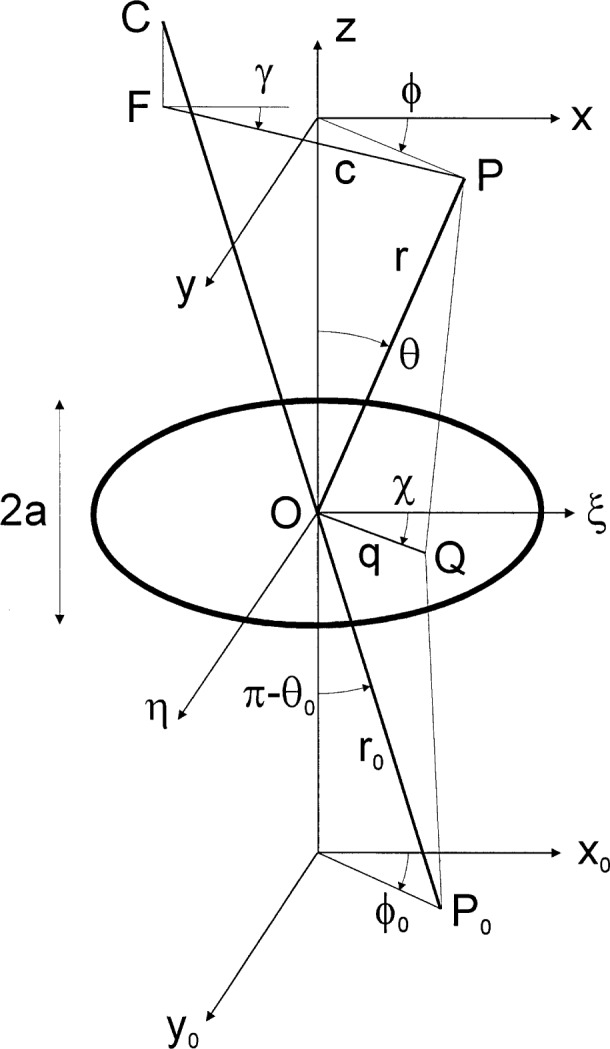
Notation for circular apertures.

A typical diffraction pattern computed in this manner is shown in Fig. 3. The numerical parameters chosen for this particular example are listed in the figure caption and were taken from an experiment described by Fresnel.^6^

## 4. Circular Aperture (Fig. 4)

### 4.1 General Theory

In evaluating the Fresnel diffraction integral Eqs. (3a–c) for a circular aperture with diameter 2*a* it is convenient to use spherical coordinates centered at the aperture center O, so that

$$\begin{array}{c} P_{0}=(x_{0},y_{0},z_{0})=r_{0}(\cos\phi_{0}\sin\theta_{0},\;\sin\phi_{0}\sin\theta_{0},\;\cos\theta_{0})\\ =-r_{0}(l_{0},m_{0},n_{0}), \end{array}$$ (13a)

Q=(ξ,η,0)=q(cosχ,sinχ,0), (13b)

$$\begin{array}{c} P=(x,y,z)=r(\cos\phi \sin\theta ,\;\sin\phi \sin\theta ,\;\cos\theta )\\ =r(l,m,n) \end{array}$$ (13c)

In these coordinates, the path difference Δ(*Q*) defined by Eq. (2e) can be evaluated as follows.

Let *C* = *r*(*l*_0_, *m*_0_, *n*_0_) be the geometrical image of *P*_0_ at the distance *r* from the aperture center, so that the position of *P* relative to *C* will be given by the vector ***CP*** = *r*(*l* − *l*_0_, *m* − *m*_0_, *n* − *n*_0_). Let *F* be the foot of the perpendicular from *C* onto the *xy*-plane, let *c* = *FP*, and define

$$\mathrm{FP}=r(l-l_{0},\;m-m_{0},\;0)=c(\cos\gamma ,\;\sin\gamma ,\;0),$$ (14a)

where

$$\begin{array}{c} c=r\sqrt{{\left(l-l_{0}\right)}^{2}+{\left(m-m_{0}\right)}^{2}}\\ =r\sqrt{\sin^{2}\theta +\sin^{2}\theta_{0}+2\sin\theta \sin\theta_{0}\cos(\phi -\phi ),} \end{array}$$ (14b)

$$\tan\gamma =\frac{m-m_{0}}{l-l_{0}}=\frac{\sin\phi \sin\theta +\sin\phi_{0}\sin\theta_{0}}{\cos\phi \sin\theta +\cos\phi_{0}\sin\phi_{0}}.$$ (14c)

Accordingly, the linear term of Eq. (2e) can be expressed in the form

$$\begin{array}{c} (l-l_{0})\xi +(m-m_{0})\eta =\frac{1}{r}\mathrm{FP}\cdot \mathrm{OQ}\\ =\frac{qc}{r}(\cos\chi \cos\gamma +\sin\chi \sin\gamma )=\frac{qc}{r}\cos(\chi -\gamma ). \end{array}$$ (14d)

In the quadratic term of Eq. (2e), we have

$$\begin{array}{c} \left(\xi^{2}+\eta^{2}\right)-{\left(l_{0}\xi +m_{0}\eta \right)}^{2}\\ =q^{2}\left[1-\sin^{2}\theta_{0}{\left(\cos\phi_{0}\cos\chi +\sin\phi_{0}\sin\chi \right)}^{2}\right]\\ =q^{2}\left[1-\sin^{2}\theta_{0}\cos^{2}\left(\chi -\phi_{0}\right)\right] \end{array}$$ (15a)

and, likewise,

$$\left(\xi^{2}+\eta^{2}\right)-{\left(l\xi +m\eta \right)}^{2}=q^{2}\left[1-\sin^{2}\theta \cos^{2}\left(\chi -\phi \right)\right].$$ (15b)

In the following, it will be assumed that the points *P*_0_ and *P* are close to the *z*-axis so that sin^2^*θ*_0_ and sin^2^*θ* are negligibly small compared to unity. In this paraxial approximation one obtains

$$\begin{array}{c} \frac{1}{2r_{0}}\left[\left(\xi^{2}+\eta^{2}\right)-{\left(l_{0}\xi +m_{0}\eta \right)}^{2}\right]\\ +\frac{1}{2r}\left[\left(\xi^{2}+\eta^{2}\right)-{\left(l\xi +m\eta \right)}^{2}\right]\approx \frac{r_{0}+r}{2r_{0}r}q^{2}. \end{array}$$ (15c)

On substitution of Eqs. (14d) and (15c) into Eq. (2e) we have

$$\Delta (Q)=-\frac{qc}{r}\cos(\chi -\gamma )+\frac{r_{0}+r}{2r_{0}r}q^{2},$$ (16a)

and hence Eq. (3c) is reduced to

$$\begin{array}{c} \alpha_{F}(P)=-\frac{ik(r_{0}+r)}{2\pi r_{0}r}\int_{0}^{a}dqq\;e^{ik\frac{r_{0}+r}{2r_{0}r}q^{2}}\int_{0}^{2\pi }d\chi e^{-ik\frac{qc}{r}\cos(\chi -\gamma )}\\ =-\frac{ik(r_{0}+r)}{r_{0}r}\int_{0}^{a}dqqJ_{0}\left(k\frac{qc}{r}\right)e^{ik\frac{r_{0}+r}{2r_{0}r}q^{2}}, \end{array}$$ (16b)

where the integral over *χ* was evaluated as 2π*J*_0_(*kqc*/*r*) [9]. On substituting

$$\rho =\frac{q}{a},\;u=\frac{ka^{2}(r_{0}+r)}{r_{0}r},\;v=\frac{kac}{r},$$ (17a)

this becomes

$$\alpha_{F}(P)=-iu\int_{0}^{1}d\rho \rho J_{0}(v\rho )e^{\frac{i}{2}u\rho^{2}}.$$ (17b)

As expected, these equations describe a circular diffraction pattern which is fully determined by a radial variable, *c* or *v*. The pattern is centered at the geometrical source image *C*, defined by *c* = *v* = 0, and *α*_F_(*P*) is constant on any circle about *C*. The radius of the geometrically illuminated spot at the distance *r* from the aperture is *a* (*r*_0_ + *r*)/*r*_0_, so that in the notation of Eq. (17a) the geometrical shadow boundary is defined by *v* = *u*. The parameter *u*, which relates the aperture radius *a* to the wavelength *λ* and the distances *r*_0_ and *r*,^7^ can assume widely different values. For example, in the case of a classroom demonstration of Fresnel diffraction, the parameters *λ* = 500 nm, *a* = 0.1 mm, *r*_0_ = *r* = 100 mm are typical and in this case one has *u* = 0.8p. On the other hand, for limiting apertures used in a radiometer, parameters such as *λ* = 500 nm, *a* = 5 mm, *r*_0_ = *r* = 1 m are typical, and then one has *u* = 200π. As will be shown later, the diffraction patterns encountered in these different cases are very different. For *u* → 0 the diffraction pattern approaches the Fraunhofer limit (Airy function), and for *u* → ∞ it approaches the limit of geometrical optics (rectangle function). (See Sec. 4.2, Figs. 6a–d).

### 4.2 Lommel’s Solution

Lommel [2] evaluated the integral Eq. (17b) in the form^8^

$$\int_{0}^{1}d\rho \rho J_{0}(v\rho )e^{\frac{i}{2}u\rho^{2}}=\frac{1}{2}[L(u,v)+\text{iM}(u,v)],$$ (18a)

so that

$$\alpha_{F}(u,v)\equiv \alpha_{L}(u,v)=\frac{u}{2}M(u,v)-i\frac{u}{2}L(u,v).$$ (18b)

In this notation,

$${\left|\alpha_{L}(u,v)\right|}^{2}=\frac{u^{2}}{4}[M^{2}(u,v)+L^{2}(u,v)]$$ (18c)

is the relative irradiance of the diffracted light and

ΦL(u,v)=arctanIm(αL)Re(αL)=−arctanL(u,v)M(u,v) (18d)

is the phase difference relative to the geometric field.

The functions L(*u*, *v*) and M(*u*, *v*) appearing in these equations are defined by

$$\begin{array}{c} \frac{u}{2}L(u,v)=\sin\frac{v^{2}}{2u}+V_{0}(u,v)\sin\frac{u}{2}-V_{1}(u,v)\cos\frac{u}{2}\\ =U_{1}(u,v)\cos\frac{u}{2}+U_{2}(u,v)\sin\frac{u}{2}, \end{array}$$ (19a)

$$\begin{array}{c} \frac{u}{2}M(u,v)=\cos\frac{v^{2}}{2u}+V_{0}(u,v)\cos\frac{u}{2}-V_{1}(u,v)\sin\frac{u}{2}\\ =U_{1}(u,v)\sin\frac{u}{2}-U_{2}(u,v)\cos\frac{u}{2}, \end{array}$$ (19b)

where

$$V_{0}(u,v)=J_{0}(v)-\left(\frac{v}{u}\right){\;}^{2}J_{2}(v)+\left(\frac{v}{u}\right){\;}^{4}J_{4}(v)+-\ldots ,$$ (20a)

$$V_{1}(u,v)=\left(\frac{v}{u}\right)J_{1}(v)-\left(\frac{v}{u}\right){\;}^{3}J_{3}(v)+\left(\frac{v}{u}\right){\;}^{5}J_{5}(v)+-\ldots ,$$ (20b)

$$U_{1}(u,v)=\left(\frac{u}{v}\right)J_{1}(v)-\left(\frac{u}{v}\right){\;}^{3}J_{3}(v)+\left(\frac{u}{v}\right){\;}^{5}J_{5}(v)+-\ldots ,$$ (20c)

$$U_{2}(u,v)=\left(\frac{u}{v}\right){\;}^{2}J_{2}(v)-\left(\frac{u}{v}\right){\;}^{4}J_{4}(v)+\left(\frac{u}{v}\right){\;}^{6}J_{6}(v)+-\ldots ,$$ (20d)

are Lommel functions of two variables, J*_n_*(*v*) being a Bessel function of the first kind and order *n*.

For checking the accuracy of numerical results, it is useful to note the values of these expressions in special cases:

- In the limit *u* → 0, Lommel’s equations simplify to the familiar Airy formula for Fraunhofer diffraction at a circular aperture. In this case, the entire diffraction pattern lies in the geometrical shadow and Eqs. (20c,d) are reduced to
  $$\frac{U_{1}(u,v)}{u}\to \frac{J_{1}(v)}{v},\;\frac{U_{2}(u,v)}{u}\to 0,\;\alpha_{L}(P)\to \frac{-iuJ_{1}(v)}{v},$$ (21)
  so that Airy’s formula, U(*P*) ∝ J_1_(*v*)/*v*, is obtained from Eqs. (17a) and (3a,b).
- For *v* = 0, we have
  Re[αL(u,0)]=(1−cosu2),Im[αL(u,0)]=−sinu2, (22a)
  $${\left|\alpha_{L}(u,0)\right|}^{2}=2\left(1-\cos\frac{u}{2}\right),$$ (22b)
  $$\Phi_{L}(u,0)=-\arctan\left[\frac{\sin(u/2)}{1-\cos(u/2)}\right].$$ (22c)
- For *v* = *u*, the well-known relations [9]
  $$\frac{1}{2}\cos u=\frac{1}{2}J_{0}(u)-J_{2}(u)+J_{4}(u)\ldots ,$$ (23a)
  $$\frac{1}{2}\sin u=J_{1}(u)-J_{3}(u)+J_{5}(u)\ldots ,$$ (23b)
  may be used to show that
  $$V_{0}(u,u)=\frac{1}{2}[J_{0}(u)+\cos u],\;V_{1}(u,u)=\frac{1}{2}\sin u,$$ (23c)
  $$U_{1}(u,u)=\frac{1}{2}\sin u,\;U_{2}(u,u)=\frac{1}{2}[J_{0}(u)-\cos u].$$ (23d)
  Accordingly,
  Re[αL(u,u)]=12[1−J0(u)]cosu2,
  Im[αL(u,u)]=−12[1+J0(u)]sinu2, (23e)
  $${\left|\alpha_{L}(u,u)\right|}^{2}=\frac{1}{4}[1-2J_{0}(u)\cos u+J_{0}^{2}(u)].$$ (23f)
  $$\Phi_{L}(u,u)=-\arctan\left[\frac{1+J_{0}(u)}{1-J_{0}(u)}\tan\frac{u}{2}\right]$$ (23g)

The use of Lommel’s equations for numerical computations is straightforward, provided that accurate values of the Bessel functions J*_n_*(*v*) required for Eqs. (20a–d) are available and the convergence behavior of these equations is taken into consideration.

When *v*/*u* or *u*/*v* are small, these expansions will converge on account of the monotonic decrease of (*v*/*u*)*^n^* or (*u*/*v*)*^n^*, provided of course that L and M are evaluated in terms of the Lommel functions V_0_ and V_1_ when *v* < *u* and in terms of U_1_ and U_2_ when *v* > *u*.

**Fig. 5:**
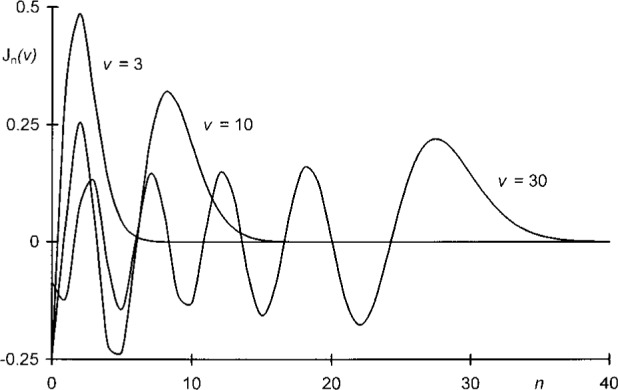
Dependence of Bessel functions J*_n_*(*v*) on *n*.

When *v*/*u* or *u*/*v* are close to unity, Eqs. (20a–d) will converge on account of the relation J*_n_*(*v*) → 0 when *n* → ∞. The manner in which this limit is approached is illustrated in Fig. 5. As *n* increases, J*_n_*(*v*) exhibits an oscillatory behavior before vanishing after passing through a pronounced final maximum at or below *n* = *v*. In this case, L and M can be evaluated in terms V_0_, V_1_ or U_1_,U_2_, although for consistency it is better to use the former method when *v* < *u* and the latter method when *v* > *u*.

It follows that, for computations of the Lommel functions to *m* decimals, the expansions Eqs. (20a–d) can be truncated when either of the conditions,

$${\left(\frac{v}{u}\right)}^{n}\text{or}{\left(\frac{u}{y}\right)}^{n}<\frac{1}{2}10^{-m},$$ (24a)

$$n\geq v\;{\text{and J}}_{n}(v)<\frac{1}{2}10^{-m},$$ (24b)

are satisfied.

**Fig. 6:**
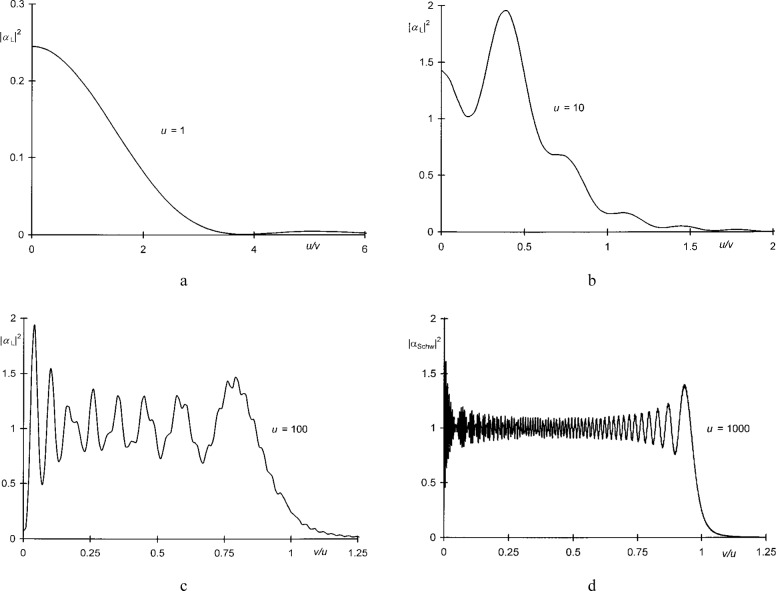
Relative irradiances for circular apertures: a, b, c) |*α*_L_(*u*, *v*)|^2^ vs *v*/*u* for *u* = 1, 10, and 100 according to Sec. 4.2. d) |*α*_Schw_(*u*, *v*)|^2^ vs *v*/*u u* for *u* = 1000 according to Sec. 4.3.

The numerical results presented in this paper were obtained on a personal computer, using standard spreadsheet software^9^ (a 133 Mhz Pentium computer and Microsoft Excel 7.0). It was found that this software provides accurate values of the Bessel functions J*_n_*(*v*) needed for Eqs. (20a–d) without problems, but that the large number of them required to satisfy Eq. (24b) impeded the speed of program execution when *u* is large and *v* ≈ *u*. In addition, the capabilities of the personal computer were overtaxed by the fact that the diffraction patterns for large values of *u* are highly structured (see Figs. 6a–d), so that a very large number of data points had to be computed. For these reasons, Eqs. (18a) to (20d) were used in this work only for *u* ≤ 300 while for larger values of the approximation of Sec. 4.3, below, was used. It should be emphasized that this limitation is unnecessary for larger computers. When sufficient computing power is available, the Lommel functions for large values of *u* can be evaluated efficiently by iterative use of recurrence relations for Bessel functions, beginning at the required large orders and iterating towards J_0_(*v*) from above, as mentioned by Shirley and Datla [13]. Under these conditions, the fine structure of the diffraction pattern poses no difficulties.

The algorithm used in this work for *u* ≤ 300 was as follows.

1. Define the value of *u*, a maximum value *v*_max_, a step size Δ*v*, and the desired decimal accuracy 10^−^*^m^*.
2. Let *v* = 0. Compute *α*_L_(*u*, 0) from Eq. (22a).
3. Let *v* = *v* + Δ*v*. Compute (*u*/2) V_0_(*u*, *v*), (*u*/2)·V_1_(*u*, *v*) from Eq. (20a,b), terminating when Eq. (24a or b) are satisfied. Compute L(*u*, *v*), M(*u*, *v*) from Eq. (19a,b) and *α*_L_(*u*, *v*) from Eq. (18b).
4. If *v* < *u*, go to Step 3.
5. Compute *α*_L_(*u*, *u*) from Eq. (23e).
6. Let *v* = *v* + Δ*v*. Compute (*u*/2) U_1_(*u*, *v*), (*u*/2) U_2_(*u*, *v*) from Eq. (20c,d), terminating when Eq. (24a or b) are satisfied. Compute L(*u*, *v*), M(*u*, *v*) from Eq. (19a,b) and *α*_L_(*u*, *v*) from Eq. (18b).
7. If *v* < *v*_max_, go to Step 6. Else, stop.

The relative irradiances |*α*_L_(*u*, *v*)|^2^ computed in this manner for *u* = 1, 10, and 100 are plotted in Figs. 6a–c.

### 4.3 Schwarzschilds’s Approximation

The above-mentioned computational problems encountered with Lommel’s solution when *u* is large can be avoided by using an asymptotic approximation for *α*_F_(*P*) derived by Schwarzschild [8] in a paper on diffraction effects in defocused telescopes. As this paper is no longer readily available, its contents will be outlined here.

Schwarzschild considered the integral

$$\begin{array}{l} W=\frac{iu}{2\pi }\int_{0}^{1}d\rho \rho \int_{0}^{2\pi }d\chi e^{-i(u\rho^{2}/2-v\rho \cos\chi +v^{2}/2u)}\\ =\frac{iu}{2\pi }\left[\int_{0}^{\infty }d\rho \ldots -\int_{1}^{\infty }d\rho \ldots \right]=W_{1}-W_{2}, \end{array}$$ (25a)

so that his approximation of Eq. (17b) is given by^10^

$$\alpha_{L}(u,v)\approx \alpha_{\text{Schw}}(u,v)=e^{\frac{iy^{2}}{2u}}W*.$$ (25b)

The integral *W*_1_ is readily shown to be equal to

$$W_{1}=1,$$ (26a)

and by evaluating the *x* -integral of *W*_2_ as in Eq. (16b) and then substituting the asymptotic expression [9]

$$J_{0}(v\rho )=\sqrt{\frac{2}{\pi \rho v}}\cos\left(v\rho -\frac{\pi }{4}\right),\;v\rho >>1$$ (26b)

one obtains

$$\begin{array}{c} W_{2}\approx \frac{1}{\sqrt{2\pi v}}[e^{\frac{i\pi }{4}}iu\int_{1}^{\infty }d\rho \sqrt{\rho }e^{-\frac{iu}{2}{\left(\rho +v/u\right)}^{2}}\\ +e^{-\frac{i\pi }{4}}iu\int_{1}^{\infty }d\rho \sqrt{\rho }e^{-\frac{iu}{2}{\left(\rho -v/u\right)}^{2}}]. \end{array}$$ (26c)

Letting

$$f=\frac{\sqrt{\rho }}{\rho +v/u},\;g=-e^{-\frac{iu}{2}{\left(\rho +v/u\right)}^{2}},$$ (27a)

the first integral in Eq. (26c) can be expressed in the form ∫*f* d*g* so that

$$\begin{array}{l} iu\int_{1}^{\infty }d\rho \sqrt{\rho }e^{-\frac{iu}{2}{\left(\rho +v/u\right)}^{2}}=\frac{e^{-\frac{i{\left(u+v\right)}^{2}}{2u}}}{1+v/u}\\ +\int_{1}^{\infty }d\left(\frac{\sqrt{\rho }}{\rho +v/u}\right)e^{-\frac{iu}{2}{\left(\rho +v/u\right)}^{2}}. \end{array}$$ (27b)

The second integral in Eq. (26b) can be written as

$$\begin{array}{c} iu\int_{1}^{\infty }d\rho \sqrt{\rho }e^{-\frac{iu}{2}{\left(\rho -v/u\right)}^{2}}\\ =iu\int_{1}^{\infty }d\rho (\sqrt{\rho }-\sqrt{v/u})e^{-\frac{iu}{2}{\left(\rho -v/u\right)}^{2}}\\ +i\sqrt{uv}\int_{1}^{\infty }d\rho e^{-\frac{iu}{2}{\left(\rho -v/u\right)}^{2}} \end{array}$$ (28a)

where the first term can again be evaluated by partial integration, using

$$f=\frac{\sqrt{\rho }-\sqrt{v/u}}{\rho -v/u}=\frac{1}{\sqrt{\rho }+\sqrt{v/u}},$$ (28b)

and the second term is a complex Fresnel integral [Eq. (8)]. In this manner, Schwarzschild found

$$\begin{array}{c} iu\int_{1}^{\infty }d\rho \sqrt{\rho }e^{-\frac{iu}{2}{\left(\rho -v/u\right)}^{2}}\\ =i\sqrt{\pi v}\{F*(\infty )-F*[\sqrt{u/\pi }(1-v/u)]\}\\ +\frac{e^{-\frac{i{\left(u-v\right)}^{2}}{2u}}}{1+\sqrt{v/u}}+\int_{1}^{\infty }d\left(\frac{1}{\sqrt{\rho }+\sqrt{v/u}}\right)e^{-\frac{iu}{2}{\left(\rho -v/u\right)}^{2}}. \end{array}$$ (28c)

He noted that, by further partial integrations, the resulting expression for *W*_2_ would become an asymptotic expansion in negative powers of *u* but that there was no point in taking the trouble as the last terms in Eqs. (27b) and (28c) are already negligibly small for practical purposes. Therefore,

$$\begin{array}{c} W=1-W_{2}\approx \frac{1}{2}+\frac{i}{\sqrt{2}}e^{-\frac{i\pi }{4}}F*[\sqrt{u/\pi }(1-v/u)]\\ -\frac{1}{\sqrt{2\pi v}}\left[\frac{e^{-\frac{i{\left(u+v\right)}^{2}}{2u}+\frac{i\pi }{4}}}{1+v/u}+\frac{e^{-\frac{i{\left(u-v\right)}^{2}}{2u}-\frac{i\pi }{4}}}{1+\sqrt{v/u}}\right],u,v>>1. \end{array}$$ (29)

Schwarzschild estimated that this expression is accurate to 0.005 if *u* = 100, *v*/*u* > 0.2; or *u* = 300, *v*/*u* > 30.

When put in the form of Eq. (25b), Schwarzschild’s approximation becomes

$$\begin{array}{l} \alpha_{F}(u,v)\approx \alpha_{\text{Schw}}(u,v)=\frac{1}{2}e^{-i\delta }-\frac{i}{\sqrt{2}}e^{-i(\delta -\pi /4)}F(s)\\ -\frac{1}{\sqrt{2\pi v}}\left[\frac{e^{i(\beta_{+}-\pi /4)}}{1+v/u}+\frac{e^{i(\beta_{-}+\pi /4)}}{1+\sqrt{v/u}}\right],u,v>>1, \end{array}$$ (30a)

Re[αSchw(u,v)]=12cosδ−12[C(s)sin(δ−π/4)−S(s)cos(δ−π/4)]−12πv[cos(β+−π/4)1+v/u+cos(β−+π/4)1+v/u],u,v>>1, (30b)

Im[αSchw(u,v)]=−12sinδ−12[C(s)cos(δ−π/4)+S(s)sin(δ−π/4)]−12πv[sin(β+−π/4)1+v/u+sin(β−+π/4)1+v/u],u,v>>1.., (30c)

where

$$\delta =\frac{v^{2}}{2u},\beta_{\pm }=\frac{u}{2}\pm v,s=\sqrt{u/\pi }(1-v/u).$$ (30d)

The use of these expressions on a computer is simple. The only caveat is that they are not valid for small values of *v*/*u* so that Lommel’s equations must still be used below a suitably chosen minimum value *v* = *v*_min_. The choice of *v*_min_ can be based on the following table, which was obtained by computing the residuals Δ = |*α*_Schw_|^2^ − |*α*_L_|^2^ for selected values of *u*.

$$\begin{array}{ccc} u & \Delta \leq 0.01 & \Delta \leq 0.001\\ 30 & \text{if}\;v/u\geq 0.23 & \text{if}\;v/u\geq 0.7\\ 100 & \text{if}\;v/u\geq 0.06 & \text{if}\;v/u\geq 0.27 \end{array}$$

For such small values of *v*/*u*, only a few terms of Eqs. (20a,b) are sufficient to obtain *α*_L_ with comparable accuracy. Thus, the following procedure will provide the entire diffraction pattern.

1. Define *u*, *v*_min_, *v*_max_, and Δ*v*.
2. Let *v* = 0. Compute *α*_L_(0, *v*) from Eq, (22a).
3. Let *v* = *v* + Δ*v*. Compute (*u*/2)·V_0_(*u*, v), (*u*/2)·V_1_(*u*, *v*) from Eqs. (20a,b), terminating after the third terms. Compute L(*u*, *v*), M(*u*, *v*) from Eqs. (19a,b) and *α*_L_(*u*, *v*) from Eq. (18b).
4. If *v* < *v*_min_, go to Step 3.
5. Let *v* = *v* + Δ*v*. Compute *δ*, *β*_±_, *s* from Eq. (30d). Use the algorithm of Sec. 3.1 to find *C*(*s*), *S*(*s*). Compute *α*_Schw_(*u*, *v*) from Eqs. (30b,c).
6. If *v* < *v*_max_, go to Step 5. Else, stop.

The values of |*α*_Schw_(*u*, *v*)|^2^ computed by this algorithm for *u* = 1000 are shown in Fig. 6d. They were found to be in excellent agreement with the values of |*α*_L_(*u*, *v*)|^2^ obtained from Lommel’s solution.^11^

**Fig. 7:**
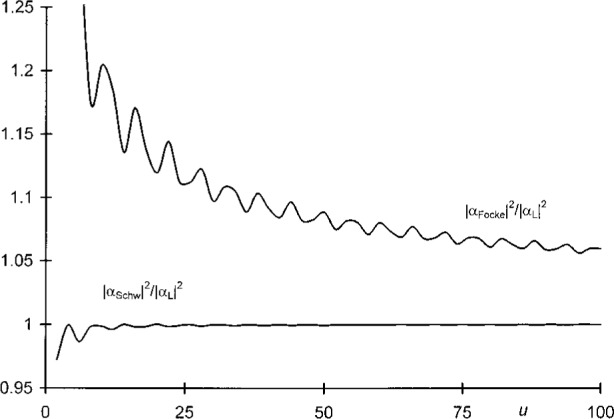
Irradiance ratios, |*α*_Schw_(*u*, *u*)|^2^/|*α*_L_(*u*, *u*)|^2^ and |*α*_Focke_(*u*, *u*)|^2^/|*α*_L_(*u*, *u*)|^2^, vs *u*.

It should be emphasized that Schwarzschild’s approximation is different from a superficially similar but significantly less accurate asymptotic approximation of the diffraction integral (17b) cited by Focke [7] and used by Blevin [14], Steel, De, and Bell [16], and Boivin [16] in their work on diffraction errors in radiometry. The respective accuracies of the Schwarzschild and Focke approximations can be assessed by comparing the relative irradiances at the shadow boundary,^12^

$$\begin{array}{c} {\left|\alpha_{\text{Schw}}(u,u)\right|}^{2}=\frac{1}{4}[1-\sqrt{\frac{8}{\pi u}}\cos u\cos\left(u-\frac{\pi }{4}\right)\\ +\frac{2}{\pi u}\cos^{2}\left(u-\frac{\pi }{4}\right)], \end{array}$$ (31a)

$${\left|\alpha_{\text{Focke}}(u,u)\right|}^{2}=\frac{1}{4}\left[1-\frac{1}{\sqrt{\pi u}}(\cos2u+\sin2u)+\frac{1}{2\pi u}\right],$$ (31b)

to the exact value |*α*_L_(*u*, *u*)|^2^ given by Eq. (23f). From the ratios plotted in Fig. 7 it follows that the Schwarzschild values are accurate to 0.1 % for *u* > 10, while for *u* = 100 the Focke values are still off by 6 %.

## Footnotes

1 In this paper it is necessary to distinguish between the “Fresnel diffraction integral” *U*_F_(*P*) defined by Eqs. (3a–c) of this paper and the “complex Fresnel integral” *F*(*s*) defined by Eq. (8).

2 A subscripted notation for wave amplitudes is used to avoid confusion between quantities which differ in physical significance and dimension. For example, the squared amplitude |*A*_sph_|^2^ of a spherical wave denotes a radiant intensity whereas the squared amplitude |*A*_plane_|^2^ of a plane wave denotes an irradiance.

3 The angle *θ*_0_ should not be confused with the angle *π* – *θ*_0_ indicated in Figs. 1 and 4. *θ* and *θ*_0_ are colatitudes which are measured clockwise from the positive *z*-axis. In this paper, *θ*_0_ is assumed to be on the order of *π*, so that cos*θ*_0_ is on the order of −1.

4 The direction cosines are identically equal to zero, and Eq. (4b) holds exactly, for the rectangular aperture discussed in Sec. 3.1.2. See Eqs. (9a–d).

5 This assumption seems to violate the condition that the values of *η* in Eq. (2e) must be small, but is justifiable on account of Fresnel’s zone construction. Because the field at *P* is not affected by zones located at large distances from the line *P*_0_*P* in Fig. 1, the *η*-integration can be extended to infinity without introducing an error.

6 Reference [1], pp. 117 and 128. At first glance, the variation of |*α*_F_(*P*)|^2^ shown in Fig. 3 does not appear to match the results described by Fresnel. However, the agreement is satisfactory when the data are replotted on a logarithmic scale to simulate Fresnel’s visual readings.

7 It should be noted that, according to Eq. (13c), the distance *r* = *z*/cos*θ* depends on the location of the point of observation *P* so that, strictly speaking, *u* is not a constant if the diffraction pattern is observed at at fixed distance *z* from the aperture. This dependence of *u* on *P* is considered negligible in the Fresnel approximation.

8 Elsewhere in the literature, L(*u*, *v*) and M(*u*, *v*) are denoted by C(*u*, *v*) and S(*u*, *v*). This practice is not followed here in order to avoid confusion with the Fresnel integrals C(*s*) and S(*s*) of Eq. (8).

9 Certain commercial equipment, instruments, or materials are identified in this paper to foster understanding. Such identification does not imply recommendation or endorsement by the National Institute of Standards and Technology, nor does it imply that the materials or equipment identified are necessarily the best available for the purpose.

10 It is well known that the wave functions for diffraction with and without a lens are complex conjugates.

11 This comparison was kindly performed by Dr. Eric Shirley of NIST.

12 Equations (31a,b) follow from Eq. (30a) of this paper and Eq. (7) of Ref. [13], respectively.
